# Antipsychotic Drug Aripiprazole Protects Liver Cells from Oxidative Stress

**DOI:** 10.3390/ijms23158292

**Published:** 2022-07-27

**Authors:** Barbara Kramar, Tinkara Pirc Marolt, Maria Monsalve, Dušan Šuput, Irina Milisav

**Affiliations:** 1 University of Ljubljana, Faculty of Medicine, Institute of Pathophysiology, Zaloska 4, SI-1000 Ljubljana, Slovenia; barbara.kramar@mf.uni-lj.si (B.K.); tinkara.pircmarolt@mf.uni-lj.si (T.P.M.); dusan.suput@mf.uni-lj.si (D.Š.); 2 Instituto de Investigaciones Biomédicas Alberto Sols (CSIC-UAM), Arturo Duperier, 4, 28029 Madrid, Spain; mpmonsalve@iib.uam.es; 3 University of Ljubljana, Faculty of Health Sciences, Laboratory of Oxidative Stress Research, Zdravstvena pot 5, SI-1000 Ljubljana, Slovenia

**Keywords:** aripiprazole, olanzapine, oxidative stress, chemical and drug induced liver injury, second-generation antipsychotics, stress response, Roussel Uclaf Causality Assessment Method (RUCAM)

## Abstract

Antipsychotics used to treat schizophrenia can cause drug-induced liver injury (DILI), according to the Roussel Uclaf Causality Assessment Method. The role of oxidative stress in triggering injury in these DILI cases is unknown. We repeatedly administrated two second-generation antipsychotics, aripiprazole and olanzapine, at laboratory alert levels to study underlying mechanisms in stress prevention upon acute oxidative stress. The drugs were administered continuously for up to 8 weeks. For this, hepatoma Fao cells, which are suitable for metabolic studies, were used, as the primary hepatocytes survive in the culture only for about 1 week. Four stress responses—the oxidative stress response, the DNA damage response and the unfolded protein responses in the endoplasmic reticulum and mitochondria—were examined in H_2_O_2_-treated cells by antioxidant enzyme activity measurements, gene expression and protein quantification. Oxidant conditions increased the activity of antioxidant enzymes and upregulated genes and proteins associated with oxidative stress response in aripiprazole-treated cells. While the genes associated with DNA damage response, Gadd45 and p21, were upregulated in both aripiprazole- and olanzapine-treated cells, only aripiprazole treatment was associated with upregulation in response to even more H_2_O_2_, which also coincided with better survival. Endoplasmic reticulum stress-induced Chop was also upregulated; however, neither endoplasmic reticulum nor mitochondrial unfolded protein response was activated. We conclude that only aripiprazole, but not olanzapine, protects liver cells against oxidative stress. This finding could be relevant for schizophrenia patients with high-oxidative-stress-risk lifestyles and needs to be validated in vivo.

## 1. Introduction

Atypical or second-generation antipsychotics (SGAs), including aripiprazole (ARI) and olanzapine (OLA), are drugs of choice for schizophrenia treatment and are effective against delusions, hallucinations, and extrapyramidal adverse events [1]. SGAs also cause adverse metabolic side effects [2], among them drug-induced liver injury (DILI) [3]. DILI associated with antidepressant use is mostly hepatocellular, while the etiology is metabolic or immuno-allergic [3]. The Roussel Uclaf Causality Assessment Method (RUCAM) is a clinical scoring method reflecting the likelihood of hepatic injury due to a specific medication [4]. Reports of RUCAM scores of SGAs are scarce; an RUCAM score of 7, indicating a probable cause of DILI, was recently reported for OLA [5]. Patients with schizophrenia and depression disorders are often treated with several antipsychotics successively or even at the same time [3,6]. This can hinder the unequivocal involvement of a particular drug in eliciting DILI.

Metabolic side-effects and unhealthy lifestyle choices of schizophrenia patients are associated with increased stress at the cellular level, often oxidative stress [7]. In experimental models, oxidative stress can induce behavioral and molecular anomalies similar to those observed in schizophrenia [8]. Indications of oxidative stress have been determined in platelets [9], serum [9] and plasma of schizophrenia patients [8], while weaker antioxidant defenses have been observed in their cerebrospinal fluids [9]. Reduced levels of antioxidant enzymes, superoxide dismutase (SOD), catalase (CAT) and glutathione peroxidase (GPx) have also been reported [8,10,11]. Information connecting psychiatric disorders and oxidative stress in patients treated with antipsychotics is therefore primarily available for the brain and blood sera [11,12,13,14,15].

Oxidative stress, an imbalance between oxidants and antioxidants, can result in liver inflammation, proliferation, and metabolic derangements [1]. Increased production of reactive oxygen/nitrogen species (ROS/RNS) can occur outside of or within cells. The most abundant ROS in animal tissues is hydrogen peroxide (H_2_O_2_), which is a proliferation stimulant at nanomolar concentrations. Micromolar levels of H_2_O_2_ cause temporal growth arrest and induce cellular protective mechanisms, such as gene expression, while millimolar levels and above instigate oxidative stress [16]. The latter induces the cell’s defense system, for example, an increase in the activity of antioxidant enzymes (superoxide dismutase (SOD), catalase (CAT), glutathione peroxidase (GPx) and glutathione) and other antioxidant compound syntheses, which act together towards balancing the ROS levels [17]. The activation of adaptive cellular responses to oxidative and other cellular stresses is therefore vital for liver injury prevention. Wink et al. [18] evaluated four adaptive cellular stress responses for the prediction of drug-induced liver injury liability by examining the key proteins: sulfiredoxin-1 (Srxn1) for oxidative stress signaling, CCAAT-enhancer-binding protein homologous protein (Chop) for the unfolded protein response (UPR) pathway, cyclin-dependent kinase inhibitor (Cdkn1a/p21) for DNA damage-related response and intercellular adhesion molecule 1 (Icam-1) for inflammatory signaling.

Here, we report on the effect of four- to eight-week ARI and OLA treatment in Fao hepatoma cells during acute oxidative stress induced by H_2_O_2_. Antioxidant enzyme induction, gene expression and protein levels of key stress-response proteins revealed antioxidant protection by ARI, but not OLA treatment. Therefore, ARI protects liver cells from acute oxidative stress better than OLA. 

## 2. Results

Rat hepatoma Fao cells were chosen instead of primary hepatocytes, as the former enable continuous drug treatment by dividing and surviving in cell cultures [19] and are also suitable for metabolic studies [20,21,22]. The concentration levels of ARI and OLA reported in patients’ plasma at (1) the laboratory alert levels and (2) a supraphysiological concentration of 6 μM were chosen in order to mimic the drugs’ influence on the patients’ hepatocytes, which are constantly exposed to these antipsychotics for long periods of time [2,23]. Cells treated with antipsychotics for 8 weeks and exposed to H_2_O_2_ for 3 h were used as a model to assess the effect of sudden oxidative stress. Since no oxidative stress responses or UPR or DNA damage responses were detected in the cells not exposed to H_2_O_2_, corresponding data are omitted from the figures, except for the control/vehicle data (i.e., baseline control, treated neither with antipsychotics nor H_2_O_2_). The experimental results are summarized in Table 1 (ARI and OLA treatments in the absence of oxidative stress are in Supplemental Figure S1).

### 2.1. Cell Survival

**Figure 1:**
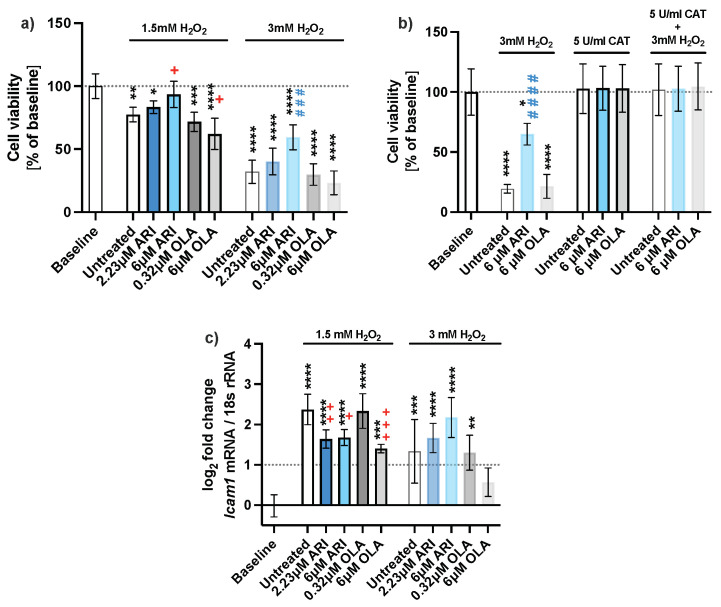
Cell viability. (**a**) Protective effect of ARI against H_2_O_2_, neutral red assay (*n* = 5), (**b**) an antioxidant enzyme catalase reverts cytotoxic effect in H_2_O_2_ treated cells (*n* = 4), and (**c**) gene expression of inflammation marker *Icam1* of long-term-treated Fao cells (*n* = 4). Data are presented as the mean ± standard deviation (SD) and analyzed with one-way ANOVA followed by the Dunnett’s test, comparing all samples to the baseline control with no added H_2_O_2_ (*); samples with 1.5 mM H_2_O_2_ added are also compared to their untreated control at 1.5 mM H_2_O_2_ (+ *p* ≤ 0.05, ++ *p* ≤ 0.01, +++ *p* ≤ 0.001); samples with 3 mM H_2_O_2_ added are compared to their untreated control at 3 mM H_2_O_2_ (### *p* ≤ 0.001, #### *p* ≤ 0.0001). * *p* ≤ 0.05, ** *p* ≤ 0.01, *** *p* ≤ 0.001, **** *p* ≤ 0.0001; *n*: number of biological replicates.

The cell viability was reduced by 40–80% when the cells were exposed to 3 mM H_2_O_2_ (Figure 1a, Supplemental Figure S2). Most cells survived in ARI-treated samples. The ARI dose-dependent increase in survival rate reached statistical significance at 6 μM ARI compared to its H_2_O_2_-treated control. In view of the observed compromised cell survival at 3 mM H_2_O_2_, we further assessed 1.5 mM H_2_O_2_ as an oxidative stress inducer. Likewise, only cells treated with 6 μM ARI were protected from oxidative stress-induced death by 1.5 mM H_2_O_2_. In fact, survival of these cells did not differ from the baseline, i.e., the untreated cells never exposed to oxidative stress. OLA-treated cells were negatively affected, implying that OLA was cytotoxic in response to oxidative stress. Therefore, although the cell response induced by 3 mM H_2_O_2_ in ARI-treated cells also supports our findings; henceforth, unless explicitly stated, we refer to the oxidative conditions induced with 1.5 mM H_2_O_2_ in the text below. Equal total RNA or protein concentrations were used among the samples for gene expression and protein measurements.

Oxidative stress due to the H_2_O_2_ treatment caused a decline in the viability of the cells (Figure 1b). An extracellular addition of an antioxidant enzyme, catalase, which neutralized H_2_O_2_, reverted the decline in all H_2_O_2_ treated cells to the level of the baseline control (dotted line). This also demonstrates that the added H_2_O_2_ is the primary source of ROS. In the absence of an antioxidant, about 3 times more ARI-treated cells survived. As the survival of OLA-treated cells was equal to the survival of untreated cells, it can be concluded that only ARI protects the cells from oxidative stress.

The inflammatory response was assessed by *Icam1* gene expression (Figure 1c), which is induced in hepatocytes in various inflammatory disorders [24,25]. There was no change in *Icam1* expression in the absence of H_2_O_2_, while there was 1.4- to 2.4-fold upregulation upon H_2_O_2_ addition. Expression of *Icam1* was the lowest in both ARI- and 6 μM OLA-treated cells, indicating the potential anti-inflammatory properties of both antipsychotic agents.

### 2.2. Oxidative Stress Response Pathway

**Figure 2:**
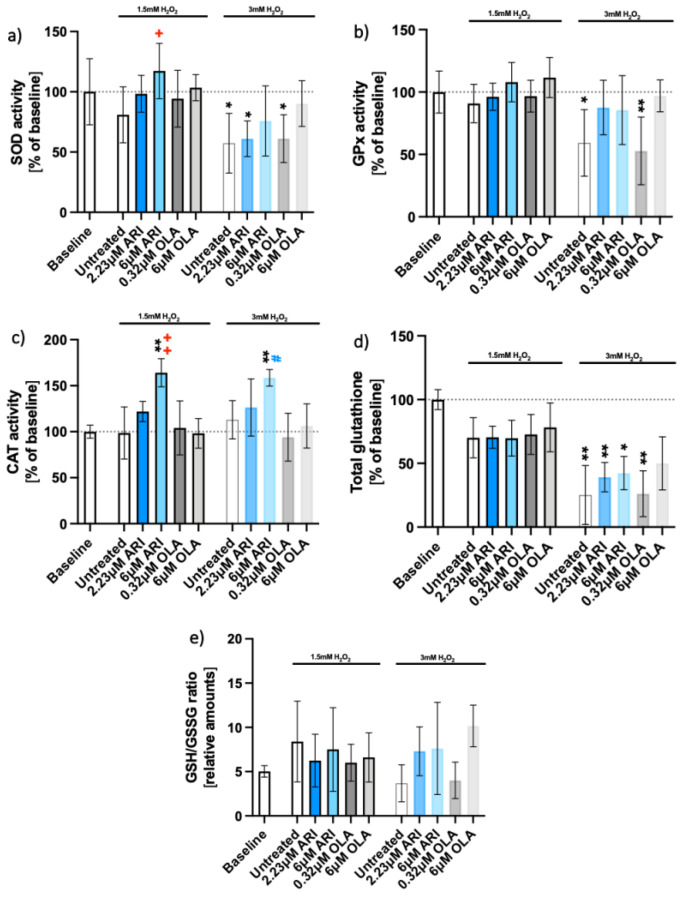
Antioxidant enzyme activities and glutathione. Enzyme activities of long-term treated Fao cells: (**a**) superoxide dismutase (SOD) activity (*n* = 5), (**b**) glutathione peroxidase (GPx) activity (*n* = 5), (**c**) catalase (CAT) activity (*n* = 4), (**d**) total glutathione (*n* = 4), and (**e**) reduced to oxidized glutathione ratio (GSH/GSSG, *n* = 3). Data are presented as the mean ± standard deviation (SD) and analyzed with one-way ANOVA followed by Dunnett’s test (**a**–**c**) or Kruskal–Wallis test followed by Dunn’s test (**d**), comparing all samples to the baseline control with no added H_2_O_2_ (*); samples with 1.5 mM H_2_O_2_ added are also compared to their untreated control at 1.5 mM H_2_O_2_ (+ *p* ≤ 0.05, ++ *p* ≤ 0.01); samples with 3 mM H_2_O_2_ added are compared to their untreated control at 3 mM H_2_O_2_ (# *p* ≤ 0.05). * *p* ≤ 0.05, ** *p* ≤ 0.01; GSH = glutathione; GSSG = glutathione disulfide; *n*: number of biological replicates.

Higher survival of ARI-treated cells was correlated with higher activities of antioxidative enzymes; both parameters were increased by ARI in a dose-dependent manner (Figure 2). The activity of SOD in 6 μM ARI-treated cells differed significantly from the untreated control in the presence of H_2_O_2_ (Figure 2a). GPx activity increased in both ARI- and 6 μM OLA-treated cells in every biological replicate, but differences did not reach statistical significance (Figure 2b). CAT activity only increased in ARI-treated cells, and the difference was statistically significant in cells treated with 6 μM ARI (Figure 2c). The total glutathione decreased upon oxidative stress exposure to a similar extent in untreated and treated cells (Figure 2d). There was no detectable difference in the reduced oxidized glutathione ratios between the drugs or concentrations used (Figure 2e). 

**Figure 3:**
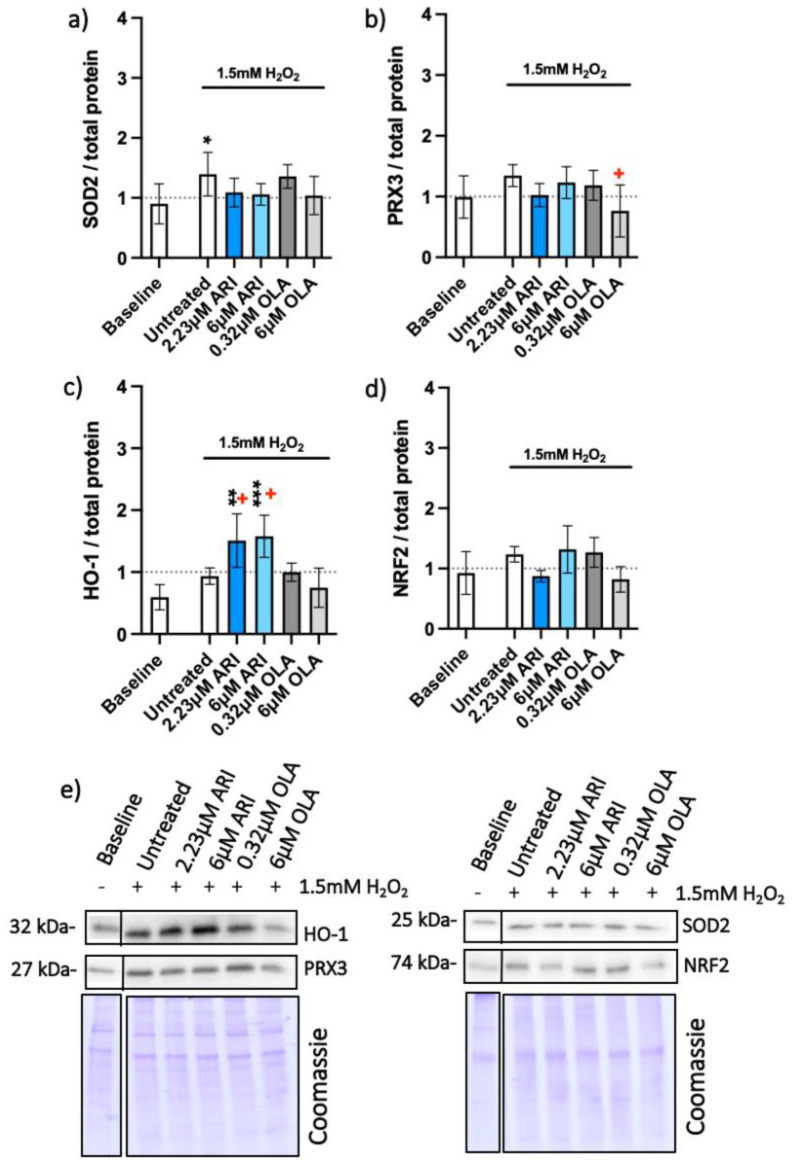
Antioxidant protein expression. Protein amounts in long-term treated Fao, standardized to total protein acquired from Coomassie-stained gels of (**a**) SOD2 (*n* = 5), (**b**) PRX3 (*n* = 5), (**c**) HO-1 (*n* = 4) and (**d**) NRF2 (*n* = 3) with (**e**) corresponding blots. Data are presented as the mean ± standard deviation (SD) and analyzed with one-way ANOVA followed by the Dunnett’s test, comparing all samples to the baseline control with no added H_2_O_2_ (*); samples with 1.5 mM H_2_O_2_ added are also compared to their untreated control at 1.5 mM H_2_O_2_ (+ *p* ≤ 0.05). * *p* ≤ 0.05, ** *p* ≤ 0.01, *** *p* ≤ 0.001; *n*: number of biological replicates.

The effect of oxidative stress on protein amounts was assessed next (Figure 3). The amounts of mitochondrial proteins superoxide dismutase 2 (SOD2) and peroxiredoxin (PRX3) in the presence of H_2_O_2_ did not statistically differ from the baseline control upon ARI treatment (Figure 2a,b). More SOD2 was observed in the untreated cells, while PRX3 was downregulated at 6 μM OLA compared to its untreated control. ARI treatment resulted in elevated heme oxygenase-1 (HO-1) compared to the baseline (Figure 3c) as well as to its untreated control, while the amounts of NRF2 protein did not differ among the groups (Figure 3d). 

**Figure 4:**
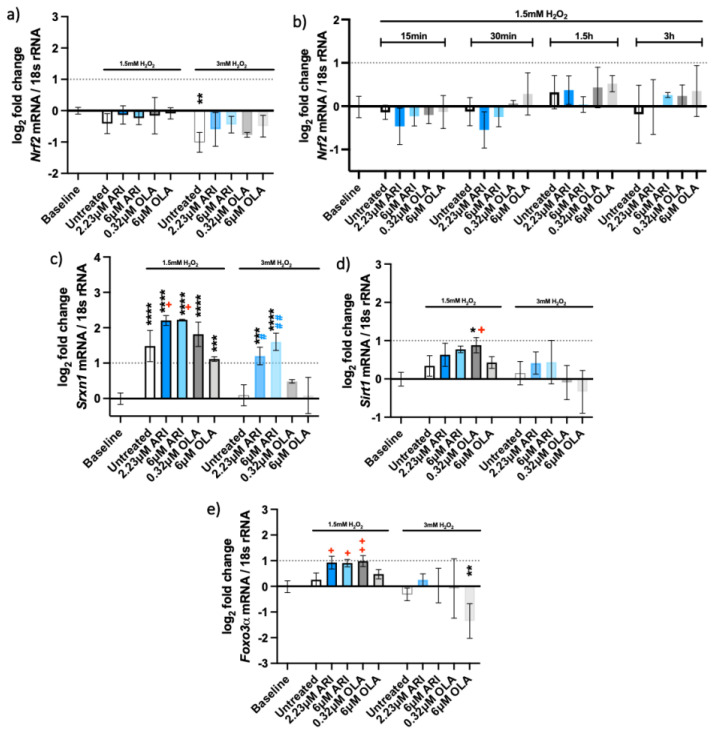
Antioxidant gene expression. Logarithmic fold-change in mRNA expression of long-term treated Fao: (**a**) *Nrf2* (*n* = 4), (**b**) *Nrf2* (single treatment for 24 h with H_2_O_2_ added as indicated, *n* = 3, (**c**) *Srxn1* (*n* = 3), (**d**) *Sirt1* (*n* = 3) and (**e**) *Foxo3*α (*n* = 3). Data are presented as the mean ± standard deviation (SD) and analyzed with one-way ANOVA followed by the Dunnett’s test, comparing all samples to the baseline control with no added H_2_O_2_ (*); samples with added 1.5 mM H_2_O_2_ are also compared to their untreated control at 1.5 mM H_2_O_2_ (+ *p* ≤ 0.05, ++ *p* ≤ 0.01); samples with added 3 mM H_2_O_2_ are compared to their untreated control at 3 mM H_2_O_2_ (# *p* ≤ 0.05, ## *p* ≤ 0.01). * *p* ≤ 0.05, ** *p* ≤ 0.01, *** *p* ≤ 0.001, **** *p* ≤ 0.0001; *n*: number of biological replicates.

Likewise, no differences in *Nrf2* gene expression were seen 3 h after H_2_O_2_ addition (Figure 4a), nor were differences measured at acute antipsychotic treatments (for 24 h) and various times of H_2_O_2_ exposure (Figure 4b). On the other hand, the gene for sulfiredoxin-1 (*Srxn-1*), whose expression is upregulated by NRF-2, e.g., during oxidative stress caused by cigarette smoke [26], was overexpressed in oxidative conditions in untreated and treated cells compared to the baseline levels (without oxidative stress). *Srxn-1* expression was higher in ARI-treated cells than in any other tested condition and this difference was statistically significant compared to the untreated cells in the presence of an oxidant (Figure 4c). These results imply that ARI treatment also increased the protection from oxidative stress by reducing the oxidized cysteines in proteins.

The expression of both *Sirt1* and *Foxo3α* was significantly higher under oxidative stress. *Sirt1* levels were slightly increased in the presence of ARI, as well as with the lower OLA concentration (Figure 4d). SIRT1 can deacetylate and activate FOXO3 in response to oxidative stress [27,28]. *Foxo3α* was significantly upregulated in the presence of ARI and the lower concentration of OLA (Figure 4e); these cells also significantly differed from the untreated ones exposed to H_2_O_2_. In conclusion, ARI likely ameliorates oxidative-stress-induced cell death by increasing antioxidative systems, as noted by the observed increases in HO-1, the upregulation of *Srxn1* and *Foxo3α*.

### 2.3. DNA Damage Response Pathway

**Figure 5:**
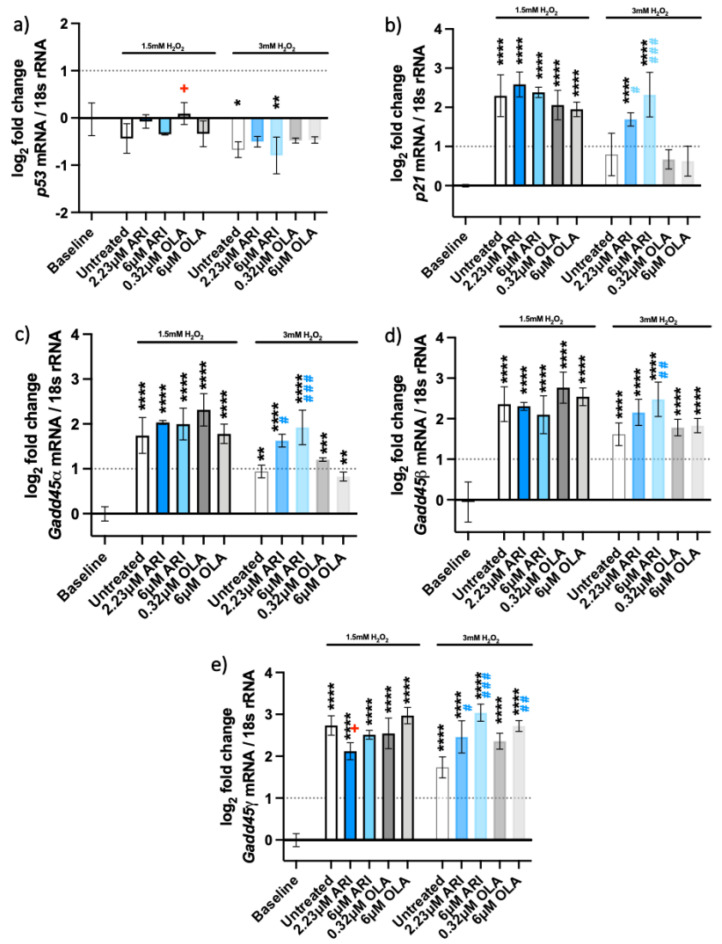
DNA damage response. Logarithmic fold-change in mRNA expression of long-term treated Fao of (**a**) *p53* (*n* = 3)*;* (**b**) *Cdkn1a/p21* (*n* = 4), (**c**) *Gadd45a* (*n* = 3), (**d**) *Gadd45β* (*n* = 4), and (**e**) *Gadd45γ* (*n* = 3). Data are presented as the mean ± standard deviation (SD) and analyzed with one-way ANOVA followed by the Dunnett’s test, comparing all samples to the baseline control with no added H_2_O_2_ (*); samples with 1.5 mM H_2_O_2_ added are also compared to their untreated control at 1.5 mM H_2_O_2_ (+ *p* ≤ 0.05); samples with 3 mM H_2_O_2_ added are compared to their untreated control at 3 mM H_2_O_2_ (# *p* ≤ 0.05, ## *p* ≤ 0.01, ### *p* ≤ 0.001). * *p* ≤ 0.05, ** *p* ≤ 0.01, *** *p* ≤ 0.001, **** *p* ≤ 0.0001; *n*: number of biological replicates.

The DNA damage response was observed upon oxidative stress exposure. We tested the expressions of five genes involved in the pathway (Figure 5), *p53* and its target genes, the cyclin-dependent kinase inhibitor 1A (*Cdkn1a/p21*) and growth arrest and DNA damage-inducible 45 (*Gadd45*) genes. We observed overexpression of *p21* (Figure 5b) and *Gadd45* genes (Figure 5c–e) in all the cells exposed to oxidative stress. The expression of the *p53* gene was not upregulated after 3 h of H_2_O_2_ treatment (Figure 5a), nor was it increased after a single antipsychotic treatment for 24 h and various H_2_O_2_ timepoints (Supplemental Figure S3). *P21* expression increased around 2-fold in response to 1.5 mM H_2_O_2_ in all conditions (Figure 5b). In contrast, this increase was only significant in the cells treated with ARI and 3 mM H_2_O_2_.

*Gadd45α*, *β* and *γ* were also 2- to 3-fold upregulated upon treatment with H_2_O_2_, implying cell stress and signaling for DNA damage repair. The expression of the three *Gadd45* genes did not differ significantly among the treatments at 1.5 mM H_2_O_2_. All the *Gadd45* genes were upregulated in ARI-treated cells exposed to 3 mM H_2_O_2_. *Gadd45γ* was also overexpressed in 6 μM OLA-treated cells and those exposed to 3 mM H_2_O_2_. The overexpression of all three *Gadd45* genes likely reflects a similar function of encoded proteins, although their induction is known to vary with the stressor and the cell type [29].

### 2.4. Unfolded Protein Response (UPR) Pathways

**Figure 6:**
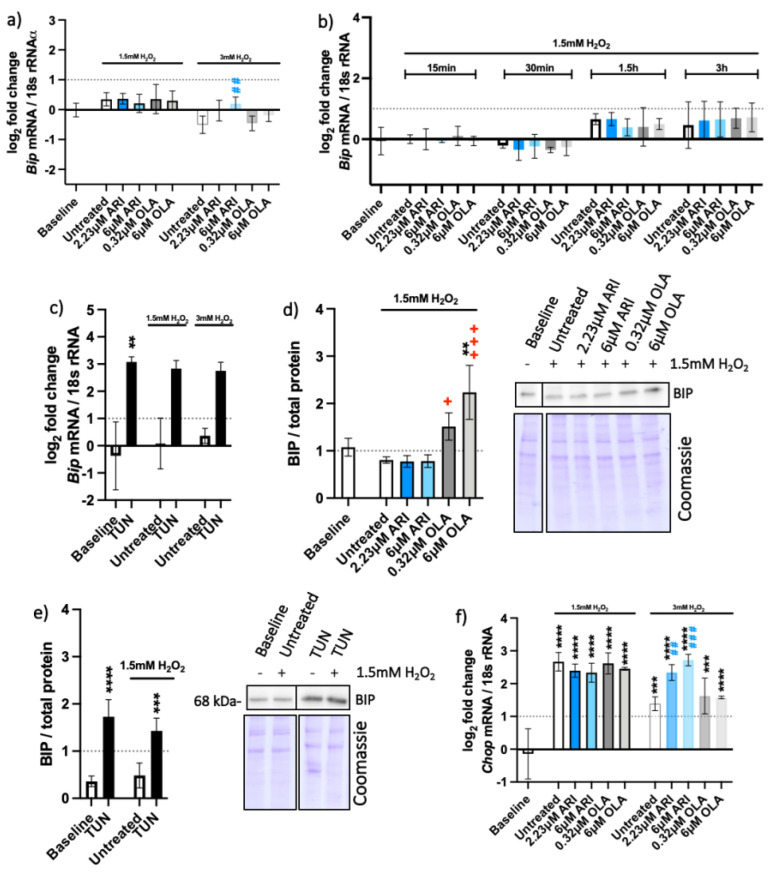
Endoplasmic reticulum UPR. Logarithmic fold-change in mRNA expression of long-term treated Fao of (**a**) *Bip* (*n* = 4)*,* (**b**) *Bip* (one-time antipsychotic treatment with H_2_O_2_ for 24 h; *n* = 3), (**c**) *Bip* (tunicamycin control, TUN; *n* = 4), and (**f**) *Ddit3*/*Chop* (*n* = 3). Protein amounts in long-term treated Fao, standardized to the total protein acquired from Coomassie-stained gels of (**d**) BIP (*n* = 3) and € BIP (tunicamycin control; *n* = 4) with corresponding blots. Data are presented as the mean ± standard deviation (SD) and for (**a**,**b**,**d**,**f**) analyzed with one-way ANOVA followed by Dunnett’s test, comparing all samples to the baseline control with no added H_2_O_2_ (*); samples with 1.5 mM H_2_O_2_ added are also compared to their untreated control at 1.5 mM H_2_O_2_ (+ *p* ≤ 0.05, +++ *p* ≤ 0.001); samples with 3 mM H_2_O_2_ added are compared to their untreated control at 3 mM H_2_O_2_ (## *p* ≤ 0.01, ### *p* ≤ 0.001). (**c**) Kruskal–Wallis test analysis with Dunn’s multiple comparison test; (**e**) one-way ANOVA analysis with Bonferroni’s multiple comparison test comparing each tunicamycin control to its untreated control. ** *p* ≤ 0.01, *** *p* ≤ 0.001, **** *p* ≤ 0.0001; *n*: number of biological replicates.

**Figure 7:**
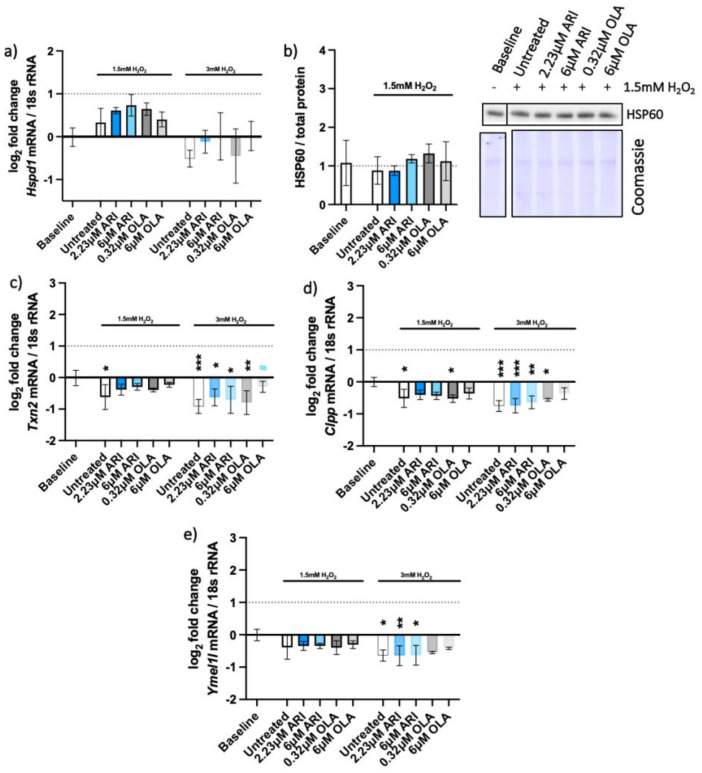
Mitochondrial UPR. Logarithmic fold-change in mRNA expression of long-term treated Fao of (**a**) *Hspd1/Hsp60* (*n* = 4), (**c**) *Txn2* (*n* = 4), (**d**) *Clpp* (*n* = 3), and (**e**) *Ymel1l* (*n* = 3); protein amounts long-term treated Fao standardized to total protein acquired from Coomassie-stained gel of (**b**) HSP60 with the corresponding blot (*n* = 4). Data are presented as the mean ± standard deviation (SD) and analyzed with one-way ANOVA followed by Dunnett’s test, comparing all samples to the baseline control with no added H_2_O_2_ (*); samples with 1.5 mM H_2_O_2_ added are also compared to their untreated control at 1.5 mM H_2_O_2_ (#). * *p* ≤ 0.05, ** *p* ≤ 0.01, *** *p* ≤ 0.001; *n*: number of biological replicates.

UPR are cellular stress responses related to the endoplasmic reticulum (erUPR) and mitochondrial (mtUPR) stress. The gene expression and protein levels of the ER chaperon-binding immunoglobulin protein (BIP) and gene expression of C/EBP Homologous Protein (*Chop*) were tested to assess the activation of erUPR (Figure 6). Gene expression and protein levels of mitochondrial heat shock protein 60 (*Hspd1* and HSP60, respectively), expression of thioredoxin 2 (*Txn2*) and protease genes caseinolytic mitochondrial matrix peptidase proteolytic subunit (*Clpp*) and YME1-like 1 ATPase (*Ymel1*) were measured to assess the mtUPR (Figure 7).

There was no substantial overexpression of the *Bip* gene in the tested cells (Figure 6a,b), although *Bip* was upregulated and resulted in increased protein amounts upon the erUPR induction with tunicamycin (Figure 6c,e). More BIP protein was found in OLA-treated cells than in any other treatment conditions (Figure 6d). BIP activation is considered one of the first signals of the erUPR and induction of CHOP is among the later ones [30]. Unlike *Bip*, the *Chop* gene was overexpressed in all cells exposed to H_2_O_2_ (Figure 6f), implying the possibility of mtUPR.

To distinguish between the two UPRs, we analyzed the expression of selected mitochondrial genes that are elevated upon the mtUPR but not erUPR (Figure 7). The pattern of HSP60 gene (*Hspd1*) expression among the differently treated cells resembled the relative amounts of the HSP60 protein on the Western blots (Figure 7a,b) and did not significantly differ among the treatments. The other mitochondrial genes that are typically upregulated upon mtUPR—thioredoxin-2 (*Txn2*) and the proteases caseinolytic mitochondrial matrix peptidase proteolytic subunit (*Clpp*) and ATP-dependent zinc metalloprotease (*Ymel1l*)—were even slightly downregulated (Figure 7c,e). These genes are typically upregulated upon the mtUPR and not erUPR, which we assessed with an erUPR trigger—tunicamycin. Accordingly, none of these genes were expressed upon the erUPR induction by tunicamycin treatment (Supplemental Figure S4).

## 3. Discussion

The lifestyle of schizophrenia patients often promotes oxidative stress, as a result of heavy smoking, infrequent bursts of physical activity, alcohol, and drug abuse [7,9,31,32,33,34]. Therefore, it is important to investigate the overall impact of oxidative stress on antipsychotics-treated liver cells, to mimic the effects of patients’ lifestyle and antipsychotic medication in the development of metabolic side-effects and DILI, which also develop in ARI-treated patients [35,36]. We focused on liver stress responses, as they can reduce oxidative damage and improve cell survival [37]. When stress responses are overwhelmed, they result in cell damage and death, which in severe cases are seen as DILI in patients. Therefore, we used previously reported markers to assess the ability of medicines to cause DILI [18] to distinguish between beneficial and overwhelmed stress responses on the way to DILI.

Cell viability, antioxidant protection, the DNA damage response, and the UPR were assessed through gene expression and protein-level measurements. To the best of our knowledge, this is the first such investigation and the first assessment of ARI in oxidant conditions.

There was no upregulation of DNA damage response and endoplasmic and mitochondrial UPR in the tested conditions. This is in agreement with the reports of no increased oxidative DNA damage in schizophrenia [9,38]. UPR induction in the endoplasmic reticulum of liver cells was reported after a single aripiprazole treatment at about 10 times higher concentrations [39]. Dose–response curves are often non-linear; therefore, the data from the two studies cannot be compared. We chose continuous treatment of cells (four to eight weeks) and used aripiprazole concentrations reported from the patients’ plasma. Therefore, our treatment regimen reflects the in vivo exposure of liver cells to these drugs.

Oxidative stress response, reported here, is concurrent with increased activity of antioxidant enzymes (SOD and CAT), higher HO-1 levels, and upregulation of *Srxn1*, *Sirt1* and *Foxo3a*. The consequence may be an observed improved survival of ARI-treated cells. Levels of antioxidant enzymes are affected in patients with schizophrenia, though the results are often inconsistent. Results from one meta-analysis point to decreased SOD activity in patients and no effect on glutathione and CAT levels [40]. On the other hand, elevated levels of CAT and GPx have also been reported [8]. The results may differ because of interindividual variability, different populations, i.e., drug-naive and medicated patients, different antipsychotics used and assessment in different tissues. Reports of lower antioxidant capacity and lower antioxidant levels in schizophrenia patients’ plasma are consistent [38,41,42,43], as well as those of weaker antioxidant defenses in their cerebrospinal fluid [9]. It would be interesting to see whether ARI has a similar antioxidative effect in other tissues as we report here in liver cells. If so, ARI may contribute to antioxidant defenses.

The ARI-induced antioxidative response that we report here in liver cells occurs only in the oxidant conditions. There is no upregulation of these genes in the absence of an acute H_2_O_2_ addition. Then, ARI slows down liver cell growth in the absence of cell death [19]. This effect is ARI specific, and was not observed with another antipsychotic, OLA. ARI improves cell survival only during acute oxidative stress. This is in agreement with the published data on preclinical models implying an increased risk of liver disease for OLA, while ARI may have protective effects [44]. Therefore, ARI can additionally protect liver cells in the presence of an oxidant, which is important, as both schizophrenia and the lifestyle choices of schizophrenia patients promote oxidative stress [7,45]. The findings of this study are encouraging and warrant validation in vivo.

## 4. Materials and Methods

All reagents were purchased from Sigma-Aldrich (Merck, St. Louis, MI, USA) unless otherwise stated.

### 4.1. Cell Cultures

The rat hepatoma cell line Fao (ECACC, 89042701) was grown at 37 °C in a humidified atmosphere with 5% CO_2_, in Coon’s F-12 Modified Liquid Medium (MBC-F0855) supplemented with 10% fetal bovine serum (Gibco, Waltham, MA, USA, 10270-106), 1% penicillin/streptomycin (Gibco, 15140-122) and 1% GlutaMAX supplement (Gibco, 35050038). The Fao cells were grown continuously with 0.12% DMSO (Acros Organics, 67-68-5), ARI (PHR1784) or OLA (PHR1825), both dissolved in 0.12% DMSO final concentration, in several parallels from different batches of cells for four weeks before they were used in experiments. ARI reaches steady-state concentrations after two weeks of administration, as reported in a pilot clinical trial [46]. Interestingly, Fao cells’ response stabilized precisely after two weeks of continuous treatment. No experimental difference was observed between four- and eight-week-treated cells. All experiments were completed after eight weeks of ARI/OLA treatment. Continuously treated cells with ARI did not grow as well as the OLA-treated cells or untreated controls [19]. As pointed out and demonstrated in our previous study, the ARI-treated cells were seeded at a higher density so that we could compare all the treatments on the same number of cells.

For cytotoxicity assessments, 40,000 cells/well were seeded in 96-well flat-bottom cell culture plates. Six-well cell culture plates (650,000 cells/well) and T25 flasks (2.4 million cells/flask) were used for gene and protein expression analyses, as well as CAT, SOD, and GPx activity measurements and determination of total glutathione.

Fao cells were grown with 0.12% DMSO as a vehicle control (“untreated” control), with concentrations of ARI and OLA equal to laboratory alert levels in patients’ sera (2.23 μM ARI and 0.32 μM OLA) and with 6 μM ARI or OLA for 24 h. Oxidative stress was induced with 1.5 mM and 3 mM H_2_O_2_ for the last 3 h of each experiment, or no H_2_O_2_ was added in the case of the baseline control.

### 4.2. Cell Viability Assay and Dehydrogenase Activity

Cytotoxicity was evaluated with 0.04 mg/mL Neutral Red dye (3-Amino-7-dimethylamino-2-methylphenazine hydrochloride; N4638) dissolved for two hours in the growth medium and washed with PBS. A reaction mix, composed of 1% glacial acetic acid (1.00063.1000) and 50% ethanol (96%, Kemika, 0505601) in water, was added to cells before the absorbance measurement at 550 nm (PerkinElmer, Victor 1420-050 spectrophotometer). Cells of one biological replicate were treated and examined simultaneously in the same microtiter plate (15 treatments in 3 technical parallels). Experiments on multiple plates were compared after dividing each value by the average of all samples of a particular replicate and data were normalized to the untreated sample without H_2_O_2_ (baseline) for presentation.

Dehydrogenase activity was measured by MTT assay as described by Pirc Marolt and colleagues [19].

### 4.3. RNA Isolation and Reverse-Transcription Quantitative Polymerase Chain Reaction Analysis (RT-qPCR)

Total RNA was isolated with TRI Reagent (T9424), its integrity checked with an Agilent 2100 Analyzer (Supplemental Figure S5) and reverse transcribed using the high-capacity cDNA reverse transcription kit (Applied Biosystems, 4368814) with added RNase inhibitor (Applied Biosystems, N8080119). PCR reactions (≤100 ng cDNA/reaction) were run in duplicates using TaqMan Universal Master Mix II, with uracil-N-glycosylase (Thermo Fisher Scientific, Waltham, MA, USA, 4440038) and quantitated using the 7500 Real-Time PCR System with SDS software (v1.3.1, Applied Biosystems, Waltham, MA, USA). SDS software was used to set the baseline and to determine the cycle threshold (CT). PCR efficiency (E) of each assay was determined using LinRegPCR software [47,48]. The expression of target genes was calculated relative to the expression of a reference gene for 18S rRNA (Rn18s) using the following equation: target/reference = (E(reference)Ct^(reference)^)/(E(target)^Ct(target)^). Biological replicates were then divided by the average of all samples in one replicate to account for multi-plate experiments and expressed as logarithmic fold change normalized to the untreated sample without H_2_O_2_ (baseline). Gene expression data were also calculated by the method published by Willems and colleagues. Data analyzed by both methods resulted in the same statistical results across all comparisons (Supplemental Figure S6) [49]. The following TaqMan probes labeled with the FAM dye (Thermo Fisher Scientific) were used: the reference gene Rn18s (Rn03928990_g1), *Nrf2/Nfe2l2* (Rn00582415_m1), *Srxn1* (Rn04337926_g1), *Sirt1* (Rn01428096_m1), *Foxo3α* (Rn01441087_m1), *Bip/Hspa5* (Rn00565250_m1), *Chop/Ddit3* (Rn00492098_g1), *p53* (Rn00755717_m1), *p21* (Rn00589996_m1), *Gadd45α* (Rn00577049_m1), *Gadd45β* (Rn01452530_g1), *Gadd45γ* (Rn01352550_g1), *Icam1* (Rn00564227_m1), *Hspd1* (Rn01441529_g1), *Txn2* (Rn00584162_g1), *Clpp* (Rn01527475_m1), *Ymel1l* (Rn00586650_m1).

### 4.4. Protein Extraction and Western Blotting

Whole-cell lysates were obtained as described by Kramar and coworkers [50], and then Western blotting was performed essentially as described by Miller and coworkers [51]. Twenty micrograms of whole-cell protein extracts were loaded onto 10 and 12% acrylamide gels to be separated by standard sodium dodecyl sulfate–polyacrylamide gel electrophoresis (SDS-PAGE) and blotted onto PVDF membranes (Immobilon-P, IPVH00010). Membranes were incubated overnight at 4 °C with primary antibodies (HO-1 (Abcam, Cambridge, UK, ab13248), Sod2 (Cell Signaling, Danvers, MA, USA, #13194), Prx3 (LabFrontiers, Koriyama, Japan, #LF-PA0030), Nrf2 (Abcam, ab31163), Bip (Cell Signaling, #3183), and Hsp60 (Abcam, ab46798)). The signal was detected by luminescence through secondary goat anti-rabbit or goat anti-mouse antibodies conjugated to horse radish peroxidase (BioRad) using X-ray films (AGFA, CP-BU, 0413), Curix60, 9462 developing machine (HO-1), or Fusion FX imager (Vilber, Marne-la-Vallée, France) (all other antibodies), and quantified by Image Studio software (LI-COR, Version 5.2.5.). Equal loadings were confirmed by staining the remaining gel with Coomassie blue dye (Fluka, 27813). The densities of sample bands were normalized to the density of the appropriate bands on the Coomassie-stained gel, and experiments on multiple blots were compared after dividing each value by the average of all the samples of a particular biological replicate.

### 4.5. Catalase, Superoxide Dismutase and Glutathione Peroxidase Activities, Total Glutathione Measurements

CAT and SOD activities were evaluated with Catalase Assay Kit (Cayman chemical, 707002) and Superoxide dismutase Assay Kit (Cayman chemical 706002), respectively, according to the manufacturer’s protocol. GPx activity and glutathione content were evaluated with Cayman Chemical Assay Kits (703102 and 703002, respectively) following the manufacturer’s instructions. Cells were harvested in 1 mM EDTA (E6758) in PBS using a cell scraper and then sonicated for 3 × 10 s on ice (cycle: 0.5, amplitude: 80%, Hielscher, UP50H) using 1 mm sonotrode (Hielscher, MS1). CAT activity was measured at 540 nm (Epoch Microplate Spectrophotometer, BioTek Instruments, Winooski, VT, USA), SOD activity at 450 nm (PerkinElmer, Victor spectrophotometer 1420–050), and GPx activity by measuring absorbance at 340 nm and glutathione at 410 nm (BioTek, Winooski, VT, United States, Epoch).

### 4.6. Statistical Analysis

Statistical analysis was performed with GraphPad Prism 9.0.0, which has an inbuilt algorithm to test the equality of variances from medians, the Brown–Forsythe test. One-way ANOVA was used in the case of equal variances, followed by Dunnett’s multiple-comparisons test (when testing multiple experimental groups against a single control group) or Bonferroni’s multiple-comparison test (when studying a relationship between variables, using thresholds based on the t-distribution), while the Kruskal–Wallis test, followed by Dunn’s multiple-comparison test was used in the case of unequal variances. Data from statistical tests (Brown–Forsythe, ANOVA, Kruskal–Wallis) are in Supplemental Tables S1–S5. Treated samples were compared to the baseline and untreated controls. All the data are presented as means ± SDs, and the significance level was set at *p* < 0.05. Statistical parameters, including the sample sizes (*n*) and p-values, are noted in the figure legends and in Table 1.

## Figures and Tables

**Table 1:** Summary statistical results with probability values (P). Gray box: no statistical significance (*p* > 0.05); green box: gene upregulation, more protein or higher enzyme activity; red box: gene downregulation, less protein, decreased cell viability or enzyme activities; white box: results are not presented/acquired due to reduced cell viability. Prot.: protein, exp.: expression, erUPR: endoplasmic reticulum unfolded protein response, mtUPR: mitochondrial unfolded protein response.

		1.5 mM H_2_O_2_ (+)						3 mM H_2_O_2_ (#)							
		ARI			OLA			ARI			OLA				
		2.23	6		0.32	6		2.23	6		0.32	6	µM		
															
**Cell viability (Figure 1)**	NR		**0.0299**			**0.0374**			**0.0008**					**↑**	Upregulation
	*Icam1*	**0.0082**	**0.0119**			**0.0008**								**↓**	Downregulation
**Antioxidant activity (Figure 2)**	SOD		**0.0298**												*p* > 0.05
	GPx														Not presented
	CAT		**0.0021**						**0.0475**						
	total GSH														
	GSH/GSSG														
**Oxidative stress—prot. exp. (Figure 3)**	SOD2														
	PRX3					**0.0118**									
	HO-1	**0.0486**	**0.0259**												
	NRF2														
**Oxidative stress—gene exp. (Figure 4)**	*Nrf2*														
	*Nrf2* (single treat.)														
	*Srxn1*	**0.0213**	**0.0183**					**0.0115**	**0.0037**						
	*Sirt1*				**0.0376**										
	*Foxo3α*	**0.0111**	**0.0134**		**0.0066**										
**DNA damage (Figure 5)**	*p53*				**0.0490**										
	*p21*							**0.0314**	**0.0006**						
	*Gadd45α*							**0.0102**	**0.0010**						
	*Gadd45β*								**0.0041**						
	*Gadd45γ*	**0.0431**						**0.0205**	**0.0005**			**0.0033**			
**erUPR (Figure 6)**	*Bip*								**0.0077**						
	*Bip* (single treat.)														
	BIP				**0.0486**	**0.0005**									
	*Chop*							**0.0041**	**0.0004**						
**mtUPR (Figure 7)**	*Hspd1*														
	HSP60														
	*Txn2*											**0.0382**			
	*Clpp*														
	*Ymel1l*														

## Data Availability

Data will be available upon acceptance from the repository of University of Ljubljana.

## Supplementary Materials

The supporting information can be downloaded at: https://www.mdpi.com/article/10.3390/ijms23158292/s1.

## References

[1] Todorovic Vukotic N., Dordevic J., Pejic S., Dordevic N., Pajovic S.B. (2021). Antidepressants- and antipsychotics-induced hepatotoxicity. Arch. Toxicol..

[2] Grajales D., Vazquez P., Ruiz-Rosario M., Tuduri E., Mirasierra M., Ferreira V., Hitos A.B., Koller D., Zubiaur P., Cigudosa J.C. (2021). The second-generation antipsychotic drug aripiprazole modulates the serotonergic system in pancreatic islets and induces beta cell dysfunction in female mice. Diabetologia.

[3] Gonzalez-Munoz M., Monserrat Villatoro J., Marin-Serrano E., Stewart S., Bardon Rivera B., Marin J., Martinez de Soto L., Seco Meseguer E., Ramirez E. (2020). A case report of a drug-induced liver injury (DILI) caused by multiple antidepressants with causality established by the updated Roussel Uclaf causality assessment method (RUCAM) and in vitro testing. Clin. Case Rep..

[4] National Institute of Diabetes and Digestive and Kidney Diseases (2012). LiverTox: Clinical and Research Information on Drug-Induced Liver Injury. Roussel Uclaf Causality Assessment Method (RUCAM) in Drug Induced Liver Injury.

[5] Brelje A., Fay B., Mariouw S., VandenBerg A. (2022). Identifying olanzapine induced liver injury in the setting of acute hepatitis C: A case report. Ment. Health Clin..

[6] Gayam V., Khalid M., Shrestha B., Hossain M.R., Dahal S., Garlapati P., Gill A., Mandal A.K., Sangha R. (2018). Drug-Induced Liver Injury: An Institutional Case Series and Review of Literature. J. Investig. Med. High. Impact Case Rep..

[7] Mahadik S.P., Evans D., Lal H. (2001). Oxidative stress and role of antioxidant and omega-3 essential fatty acid supplementation in schizophrenia. Prog. Neuropsychopharmacol. Biol. Psychiatry.

[8] Bitanihirwe B.K., Woo T.U. (2011). Oxidative stress in schizophrenia: An integrated approach. Neurosci. Biobehav. Rev..

[9] Madireddy S., Madireddy S. (2020). Regulation of Reactive Oxygen Species-Mediated Damage in the Pathogenesis of Schizophrenia. Brain Sci..

[10] Wu J.Q., Kosten T.R., Zhang X.Y. (2013). Free radicals, antioxidant defense systems, and schizophrenia. Prog. Neuropsychopharmacol. Biol. Psychiatry.

[11] Wu Z.W., Yu H.H., Wang X., Guan H.Y., Xiu M.H., Zhang X.Y. (2021). Interrelationships Between Oxidative Stress, Cytokines, and Psychotic Symptoms and Executive Functions in Patients With Chronic Schizophrenia. Psychosom. Med..

[12] Padurariu M., Ciobica A., Dobrin I., Stefanescu C. (2010). Evaluation of antioxidant enzymes activities and lipid peroxidation in schizophrenic patients treated with typical and atypical antipsychotics. Neurosci. Lett..

[13] Hendouei N., Farnia S., Mohseni F., Salehi A., Bagheri M., Shadfar F., Barzegar F., Hoseini S.D., Charati J.Y., Shaki F. (2018). Alterations in oxidative stress markers and its correlation with clinical findings in schizophrenic patients consuming perphenazine, clozapine and risperidone. Biomed. Pharmacother..

[14] Lin C.H., Lane H.Y. (2019). Early Identification and Intervention of Schizophrenia: Insight From Hypotheses of Glutamate Dysfunction and Oxidative Stress. Front. Psychiatry.

[15] Wei Z., Bai O., Richardson J.S., Mousseau D.D., Li X.M. (2003). Olanzapine protects PC12 cells from oxidative stress induced by hydrogen peroxide. J. Neurosci. Res..

[16] Pickering A.M., Vojtovich L., Tower J., KJ A.D. (2013). Oxidative stress adaptation with acute, chronic, and repeated stress. Free Radic. Biol. Med..

[17] Cichoz-Lach H., Michalak A. (2014). Oxidative stress as a crucial factor in liver diseases. World J. Gastroenterol..

[18] Wink S., Hiemstra S.W., Huppelschoten S., Klip J.E., van de Water B. (2018). Dynamic imaging of adaptive stress response pathway activation for prediction of drug induced liver injury. Arch. Toxicol..

[19] Pirc Marolt T., Kramar B., Bulc Rozman K., Suput D., Milisav I. (2020). Aripiprazole reduces liver cell division. PLoS ONE.

[20] Scarino M.L., Howell K.E. (1987). The Fao Cell. A tissue culture model for lipoprotein synthesis and secretion. Exp. Cell Res..

[21] Bayly A.C., French N.J., Dive C., Roberts R.A. (1993). Non-genotoxic hepatocarcinogenesis in vitro: The FaO hepatoma line responds to peroxisome proliferators and retains the ability to undergo apoptosis. J. Cell Sci..

[22] Monsalve M., Prieto I., de Bem A.F., Olmos Y. (2019). Methodological Approach for the Evaluation of FOXO as a Positive Regulator of Antioxidant Genes. Methods Mol. Biol..

[23] Hiemke C., Bergemann N., Clement H.W., Conca A., Deckert J., Domschke K., Eckermann G., Egberts K., Gerlach M., Greiner C. (2018). Consensus Guidelines for Therapeutic Drug Monitoring in Neuropsychopharmacology: Update 2017. Pharmacopsychiatry.

[24] Gulubova M.V. (1998). Intercellular adhesion molecule-1 (ICAM-1) expression in the liver of patients with extrahepatic cholestasis. Acta Histochem..

[25] Volpes R., van den Oord J.J., Desmet V.J. (1990). Hepatic expression of intercellular adhesion molecule-1 (ICAM-1) in viral hepatitis B. Hepatology.

[26] Singh A., Ling G., Suhasini A.N., Zhang P., Yamamoto M., Navas-Acien A., Cosgrove G., Tuder R.M., Kensler T.W., Watson W.H. (2009). Nrf2-dependent sulfiredoxin-1 expression protects against cigarette smoke-induced oxidative stress in lungs. Free Radic. Biol. Med..

[27] Brunet A., Sweeney L.B., Sturgill J.F., Chua K.F., Greer P.L., Lin Y., Tran H., Ross S.E., Mostoslavsky R., Cohen H.Y. (2004). Stress-dependent regulation of FOXO transcription factors by the SIRT1 deacetylase. Science.

[28] Wang X., Hu S., Liu L. (2017). Phosphorylation and acetylation modifications of FOXO3a: Independently or synergistically?. Oncol. Lett..

[29] Tamura R.E., de Vasconcellos J.F., Sarkar D., Libermann T.A., Fisher P.B., Zerbini L.F. (2012). GADD45 proteins: Central players in tumorigenesis. Curr. Mol. Med..

[30] Oyadomari S., Mori M. (2004). Roles of CHOP/GADD153 in endoplasmic reticulum stress. Cell Death Differ..

[31] Adamowicz K., Kucharska-Mazur J. (2020). Dietary Behaviors and Metabolic Syndrome in Schizophrenia Patients. J. Clin. Med..

[32] Simonelli-Munoz A.J., Fortea M.I., Salorio P., Gallego-Gomez J.I., Sanchez-Bautista S., Balanza S. (2012). Dietary habits of patients with schizophrenia: A self-reported questionnaire survey. Int. J. Ment. Health Nurs..

[33] Belviranlı M., Gökbel H. (2006). Acute exercise induced oxidative stress and antioxidant changes. Eur. J. Gen. Med..

[34] Fisher-Wellman K., Bloomer R.J. (2009). Acute exercise and oxidative stress: A 30 year history. Dyn. Med..

[35] Kornischka J., Cordes J. (2016). Acute Drug-Induced Hepatitis during Aripiprazole Monotherapy: A Case Report. J. Pharmacovigil..

[36] Castanheira L., Fernandes E., Levy P., Coentre R. (2019). Aripiprazole-induced Hepatitis: A Case Report. Clin. Psychopharmacol. Neurosci..

[37] Sanchez-Ramos C., Prieto I., Tierrez A., Laso J., Valdecantos M.P., Bartrons R., Rosello-Catafau J., Monsalve M. (2017). PGC-1alpha Downregulation in Steatotic Liver Enhances Ischemia-Reperfusion Injury and Impairs Ischemic Preconditioning. Antioxid. Redox Signal..

[38] Hasan Tahsin Kilic O., Aksoy I., Cinpolat Elboga G., Bulbul F. (2019). Oxidative parameters, oxidative DNA damage, and urotensin-II in schizoaffective disorder patients. Psychiatry Clin. Psychopharmacol..

[39] Forno F., Maatuf Y., Boukeileh S., Dipta P., Mahameed M., Darawshi O., Ferreira V., Rada P., Garcia-Martinez I., Gross E. (2020). Aripiprazole Cytotoxicity Coincides with Activation of the Unfolded Protein Response in Human Hepatic Cells. J. Pharmacol. Exp. Ther..

[40] Zhang M., Zhao Z., He L., Wan C. (2010). A meta-analysis of oxidative stress markers in schizophrenia. Sci. China Life Sci..

[41] Yao J.K., Reddy R., van Kammen D.P. (1998). Reduced level of plasma antioxidant uric acid in schizophrenia. Psychiatry Res..

[42] Yao J.K., Reddy R.D., van Kammen D.P. (2001). Oxidative damage and schizophrenia: An overview of the evidence and its therapeutic implications. CNS Drugs.

[43] Reddy R., Keshavan M., Yao J.K. (2003). Reduced plasma antioxidants in first-episode patients with schizophrenia. Schizophr. Res..

[44] Isaacson R.H., Beier J.I., Khoo N.K., Freeman B.A., Freyberg Z., Arteel G.E. (2020). Olanzapine-induced liver injury in mice: Aggravation by high-fat diet and protection with sulforaphane. J. Nutr. Biochem..

[45] Hursitoglu O., Orhan F.O., Kurutas E.B., Doganer A., Durmus H.T., Kopar H. (2021). Diagnostic Performance of Increased Malondialdehyde Level and Oxidative Stress in Patients with Schizophrenia. Noro Psikiyatr. Ars..

[46] Beresford T.P., Clapp L., Martin B., Wiberg J.L., Alfers J., Beresford H.F. (2005). Aripiprazole in schizophrenia with cocaine dependence: A pilot study. J. Clin. Psychopharmacol..

[47] Ramakers C., Ruijter J.M., Deprez R.H., Moorman A.F. (2003). Assumption-free analysis of quantitative real-time polymerase chain reaction (PCR) data. Neurosci. Lett..

[48] Ruijter J.M., Ramakers C., Hoogaars W.M., Karlen Y., Bakker O., van den Hoff M.J., Moorman A.F. (2009). Amplification efficiency: Linking baseline and bias in the analysis of quantitative PCR data. Nucleic Acids Res..

[49] Willems E., Leyns L., Vandesompele J. (2008). Standardization of real-time PCR gene expression data from independent biological replicates. Anal. Biochem..

[50] Kramar B., Suput D., Milisav I. (2021). Differential p16 expression levels in the liver, hepatocytes and hepatocellular cell lines. PeerJ.

[51] Miller I.P., Pavlovic I., Poljsak B., Suput D., Milisav I. (2019). Beneficial Role of ROS in Cell Survival: Moderate Increases in H_2_O_2_ Production Induced by Hepatocyte Isolation Mediate Stress Adaptation and Enhanced Survival. Antioxidants.

