# Supplementary material for: Antipsychotic Drug Aripiprazole Protects Liver Cells from Oxidative Stress
Source: Int J Mol Sci. 2022 Jul 27;23(15):8292. doi: 10.3390/ijms23158292 (PMC9368927; doi:10.3390/ijms23158292)
Supplement: Supplementary file 1 [file ijms-23-08292-s001.zip › Supp Tables.pdf]

Supplemental Table S1

a) Compared to Baseline

Fig	Brown-Forsythe test		ANOVA		Kruskal-Wallis
	**F**	**p-value**	**F**	**p-value**	**p-value**
**1a**	0.2157	0.9936	**41.99**	**<0.0001**	n.a.
**1b**	0.4370	0.9040	**16.54**	**<0.0001**	n.a.
**1c**	1.585	0.1575	**12.36**	**<0.0001**	n.a.
**2a**	0.2935	0.9792	**4.172**	**0.0004**	n.a.
**2b**	0.7591	0.6661	**4.297**	**0.0004**	n.a.
**2c**	0.8543	0.5827	**5.055**	**0.0002**	n.a.
**2d**	**3.451**	**0.0034**	n.a.	n.a.	**0.0004**
**2e**	0.3387	0.9601	1.061	0.43	n.a.
**3a**	0.2395	0.941	2.395	0.0675	n.a.
**3b**	0.4997	0.7734	2.548	0.0551	n.a.
**3c**	0.7714	0.5826	7.992	0.0004	n.a.
**3d**	0.3396	0.8792	2.189	0.1235	n.a.
**4a**	2.016	0.0661	**3.121**	**0.0072**	n.a.
**4c**	0.7400	0.6805	**25.54**	**<0.0001**	n.a.

b) Compared to Untreated control + 1.5 mM H_2O_2

Fig	Brown-Forsythe test		ANOVA		Kruskal-Wallis
	**F**	**p-value**	**F**	**p-value**	**p-value**
**1a**	0.4211	0.7915	**9.198**	**0.0002**	n.a.
**1b**	n.a.	n.a.	n.a.	n.a.	n.a.
**1c**	1.079	0.4040	**9.223**	**0.0007**	n.a.
**2a**	0.4703	0.7569	2.233	0.1018	n.a.
**2b**	0.3374	0.8493	1.738	0.1833	n.a.
**2c**	0.9624	0.4564	**6.816**	**0.0025**	n.a.
**2d**	1.528	0.2444	0.2227	0.9215	n.a.
**2e**	0.1512	0.9582	0.2293	0.9157	n.a.
**3a**	0.2694	0.8942	2.068	0.1232	n.a.
**3b**	0.5659	0.6902	**3.305**	**0.0311**	n.a.
**3d**	0.8144	0.5355	**6.17**	**0.0038**	n.a.
**3c**	0.2897	0.8781	2.952	0.0752	n.a.
**4a**	1.575	0.2321	0.5459	0.7048	n.a.
**4c**	1.151	0.3877	**10.17**	**0.0015**	n.a.

c) Compared to Untreated control + 3 mM H_2O_2

Fig	Brown-Forsythe test		ANOVA		Kruskal-Wallis
	**F**	**p-value**	**F**	**p-value**	**p-value**
**1a**	0.0393	0.9968	**10.55**	**<0.0001**	n.a.
**1b**	0.5365	0.6024	**41.18**	**<0.0001**	n.a.
**1c**	1.287	0.322	**5.126**	**0.0093**	n.a.
**2a**	0.2096	0.9301	1.951	0.1411	n.a.
**2b**	0.5480	0.7026	**3.246**	**0.0332**	n.a.
**2c**	0.6478	0.6376	**4.206**	**0.0192**	n.a.
**2d**	**6.348**	**0.0034**	n.a.	n.a.	0.4523
**2e**	0.4451	0.7738	2.260	0.1349	n.a.
**3a**	n.a.	n.a.	n.a.	n.a.	n.a.
**3b**	n.a.	n.a.	n.a.	n.a.	n.a.
**3c**	n.a.	n.a.	n.a.	n.a.	n.a.
**3d**	n.a.	n.a.	n.a.	n.a.	n.a.
**4a**	2.915	0.0602	1.686	0.2089	n.a.
**4c**	0.5119	0.7295	**10.54**	**0.0028**	n.a.

**4d**	0.5255	0.853	**3.551**	**0.0069**	n.a.
**4e**	2.039	0.0812	**7.235**	**<0.0001**	n.a.
**5a**	0.4958	0.8739	**4.063**	**0.0033**	n.a.
**5b**	0.8596	0.5786	**20.57**	**<0.0001**	n.a.
**5c**	0.7467	0.675	**21.1**	**<0.0001**	n.a.
**5d**	1.394	0.2305	**16.12**	**<0.0001**	n.a.
**5e**	0.2542	0.9848	**36.77**	**<0.0001**	n.a.
**6a**	0.2928	0.9778	**4.678**	**0.0004**	n.a.
**6d**	1.411	0.2884	**12.7**	**0.0002**	n.a.
**6f**	1.005	0.4704	**19.1**	**<0.0001**	n.a.
**7a**	1.624	0.1492	**5.971**	**<0.0001**	n.a.
**7b**	1.337	0.2936	0.8952	0.5051	n.a.
**7c**	1.186	0.3381	**3.971**	**0.0016**	n.a.
**7d**	0.2406	0.9878	**4.16**	**0.0029**	n.a.
**7e**	0.3790	0.9422	**2.384**	**0.0449**	n.a.

**4d**	0.4950	0.7401	3.311	0.0567	n.a.
**4e**	0.09567	0.9815	**6.855**	**0.0064**	n.a.
**5a**	0.6622	0.6322	2.939	0.0759	n.a.
**5b**	0.6395	0.643	2.182	0.124	n.a.
**5c**	0.7366	0.5878	1.712	0.2234	n.a.
**5d**	1.755	0.194	1.861	0.1734	n.a.
**5e**	0.2023	0.9308	**4.862**	**0.023**	n.a.
**6a**	0.3770	0.8213	0.167	0.9516	n.a.
**6d**	1.676	0.2311	**14.13**	**0.0004**	n.a.
**6f**	0.3840	0.8153	0.9931	0.4547	n.a.
**7a**	2.193	0.1267	2.243	0.1206	n.a.
**7b**	1.461	0.2631	1.616	0.2219	n.a.
**7c**	2.235	0.1175	2.015	0.1476	n.a.
**7d**	0.2896	0.8782	0.4559	0.7665	n.a.
**7e**	0.3313	0.8508	0.09058	0.9833	n.a.

**4d**	0.2027	0.9305	1.583	0.26	n.a.
**4e**	1.821	0.2091	3.108	0.0728	n.a.
**5a**	0.6755	0.6257	1.355	0.3225	n.a.
**5b**	0.8179	0.5348	**12.3**	**0.0002**	n.a.
**5c**	0.6895	0.6173	**14.48**	**0.0006**	n.a.
**5d**	2.052	0.1419	**5.137**	**0.0093**	n.a.
**5e**	0.3574	0.8328	**11.07**	**0.0016**	n.a.
**6a**	0.2261	0.9192	**4.624**	**0.0137**	n.a.
**6d**	n.a.	n.a.	n.a.	n.a.	n.a.
**6f**	2.298	0.138	**15.08**	**0.0005**	n.a.
**7a**	1.194	0.3564	1.414	0.2802	n.a.
**7b**	n.a.	n.a.	n.a.	n.a.	n.a.
**7c**	0.5302	0.7156	2.346	0.105	n.a.
**7d**	0.2755	0.8865	2.229	0.1463	n.a.
**7e**	0.6012	0.6714	0.5888	0.6792	n.a.

Supplemental Table S2

	Brown-Forsythe test		ANOVA		Kruskal-Wallis
**Fig 6**	**F**	**p-value**	**F**	**p-value**	**p-value**
**6c**	**35.53**	**<0.0001**	n.a.	n.a.	**0.0028**
**6e**	1.804	0.2001	**25.73**	**<0.0001**	n.a.

Supplemental Table S3

a) Compared to Baseline

	Brown-Forsythe test		ANOVA		Kruskal-Wallis
**Fig**	**F**	**p-value**	**F**	**p-value**	**p-value**
**4b**	0.4987	0.9532	**2.029**	**0.0248**	n.a.
**6b**	0.7234	0.7816	**3.076**	**0.0009**	n.a.

b) Compared to Untreated control + 1.5mM H_2O_2 (15min)

	Brown-Forsythe test		ANOVA		Kruskal-Wallis
**Fig**	**F**	**p-value**	**F**	**p-value**	**p-value**
**4b**	0.2767	0.8864	0.6325	0.6507	n.a.
**6b**	0.4043	0.8015	0.2603	0.8968	n.a.

c) Compared to Untreated control + 1.5mM H_2O_2 (30min)

	Brown-Forsythe test		ANOVA		Kruskal-Wallis
**Fig**	**F**	**p-value**	**F**	**p-value**	**p-value**
**4b**	0.6420	0.6447	2.601	0.1003	n.a.
**6b**	0.6050	0.668	0.1817	0.9427	n.a.

d) Compared to Untreated control + 1.5mM H_2O_2 (1.5h)

	Brown-Forsythe test		ANOVA		Kruskal-Wallis
**Fig**	**F**	**p-value**	**F**	**p-value**	**p-value**
**4b**	0.4890	0.7441	0.9247	0.4872	n.a.
**6b**	0.3803	0.8178	0.4414	0.7764	n.a.

e) Compared to Untreated control + 1.5mM H_2O_2 (3h)

	Brown-Forsythe test		ANOVA		Kruskal-Wallis
**Fig**	**F**	**p-value**	**F**	**p-value**	**p-value**
**4b**	0.5140	0.7274	0.5884	0.6787	n.a.
**6b**	0.2712	0.8899	0.08955	0.9837	n.a.

Supplemental Table S4

a) Compared to Baseline

	Brown-Forsythe test		ANOVA		Kruskal-Wallis
**Fig**	**F**	**p-value**	**F**	**p-value**	**p-value**
**S3**	0.4828	0.9601	0.6343	0.8647	n.a.

b) Compared to Untreated control + 1.5mM H_2O_2 (15min)

	Brown-Forsythe test		ANOVA		Kruskal-Wallis
**Fig**	**F**	**p-value**	**F**	**p-value**	**p-value**
**S3**	0.9249	0.4871	0.6303	0.652	n.a.

c) Compared to Untreated control + 1.5mM H_2O_2 (30min)

	Brown-Forsythe test		ANOVA		Kruskal-Wallis
**Fig**	**F**	**p-value**	**F**	**p-value**	**p-value**
**S3**	0.3927	0.8095	0.9082	0.4953	n.a.

d) Compared to Untreated control + 1.5mM H_2O_2 (1.5h)

	Brown-Forsythe test		ANOVA		Kruskal-Wallis
**Fig**	**F**	**p-value**	**F**	**p-value**	**p-value**
**S3**	0.5195	0.7237	0.6449	0.6429	n.a.

e) Compared to Untreated control + 1.5mM H_2O_2 (3h)

	Brown-Forsythe test		ANOVA		Kruskal-Wallis
**Fig**	**F**	**p-value**	**F**	**p-value**	**p-value**
**S3**	0.1602	0.9537	0.9897	0.4563	n.a.

Supplemental Table S5

	Brown-Forsythe test		ANOVA		Kruskal-Wallis
**Fig**	**F**	**p-value**	**F**	**p-value**	**p-value**
**S4a**	**3.420**	**0.024**	n.a.	n.a	0.5057
**S4b**	**3.780**	**0.0163**	n.a.	n.a.	**0.0026**
**S4c**	0.2839	0.913	**3.198**	**0.0459**	n.a.
**S4d**	0.2273	0.9435	**2.022**	**0.1475**	n.a.
