# Supplementary figures and images for: Antipsychotic Drug Aripiprazole Protects Liver Cells from Oxidative Stress
Source: Int J Mol Sci. 2022 Jul 27;23(15):8292. doi: 10.3390/ijms23158292 (PMC9368927; doi:10.3390/ijms23158292)

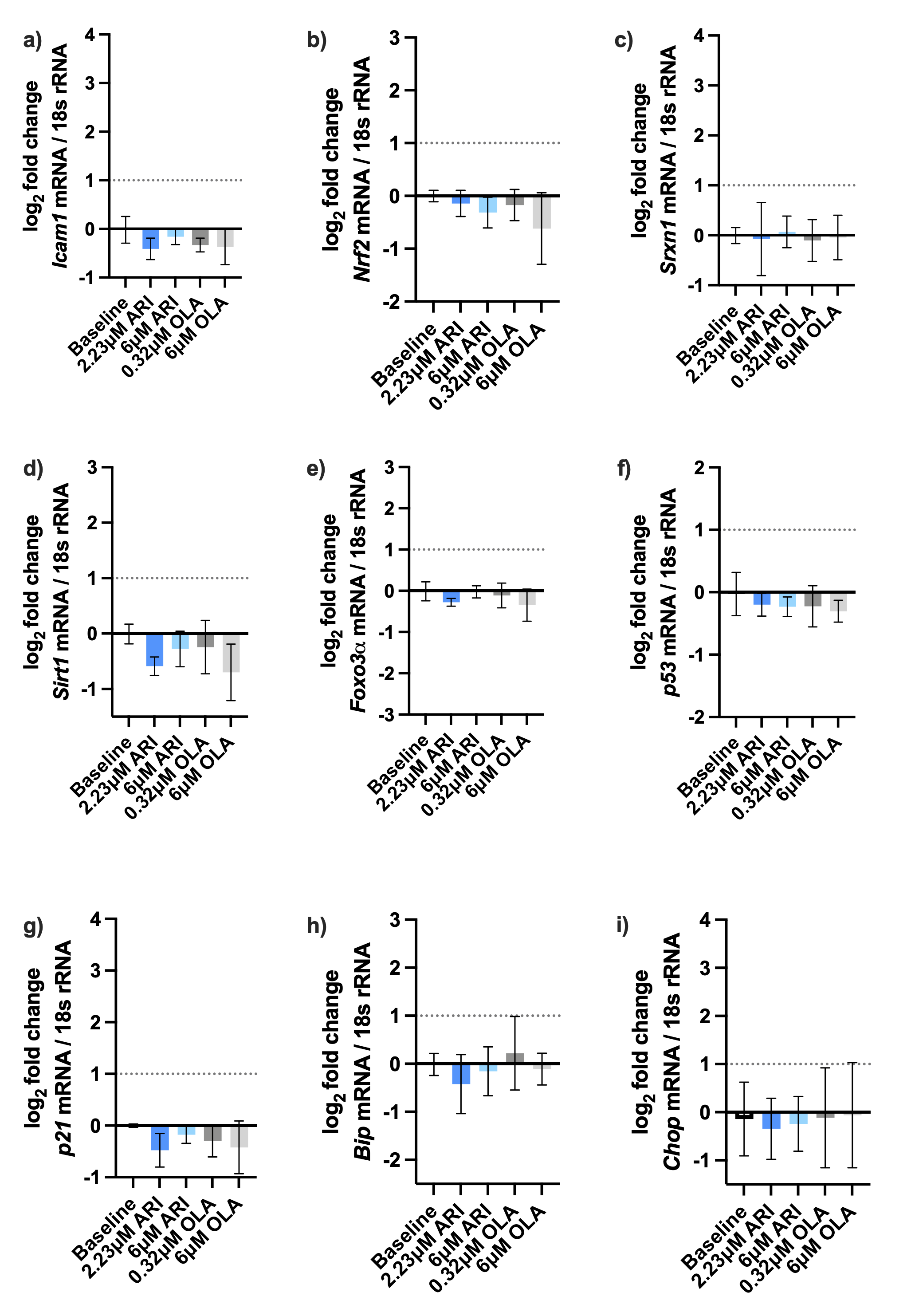

Supplement: Supplementary file 1 [file ijms-23-08292-s001.zip › Suppl Figure S1.tiff]

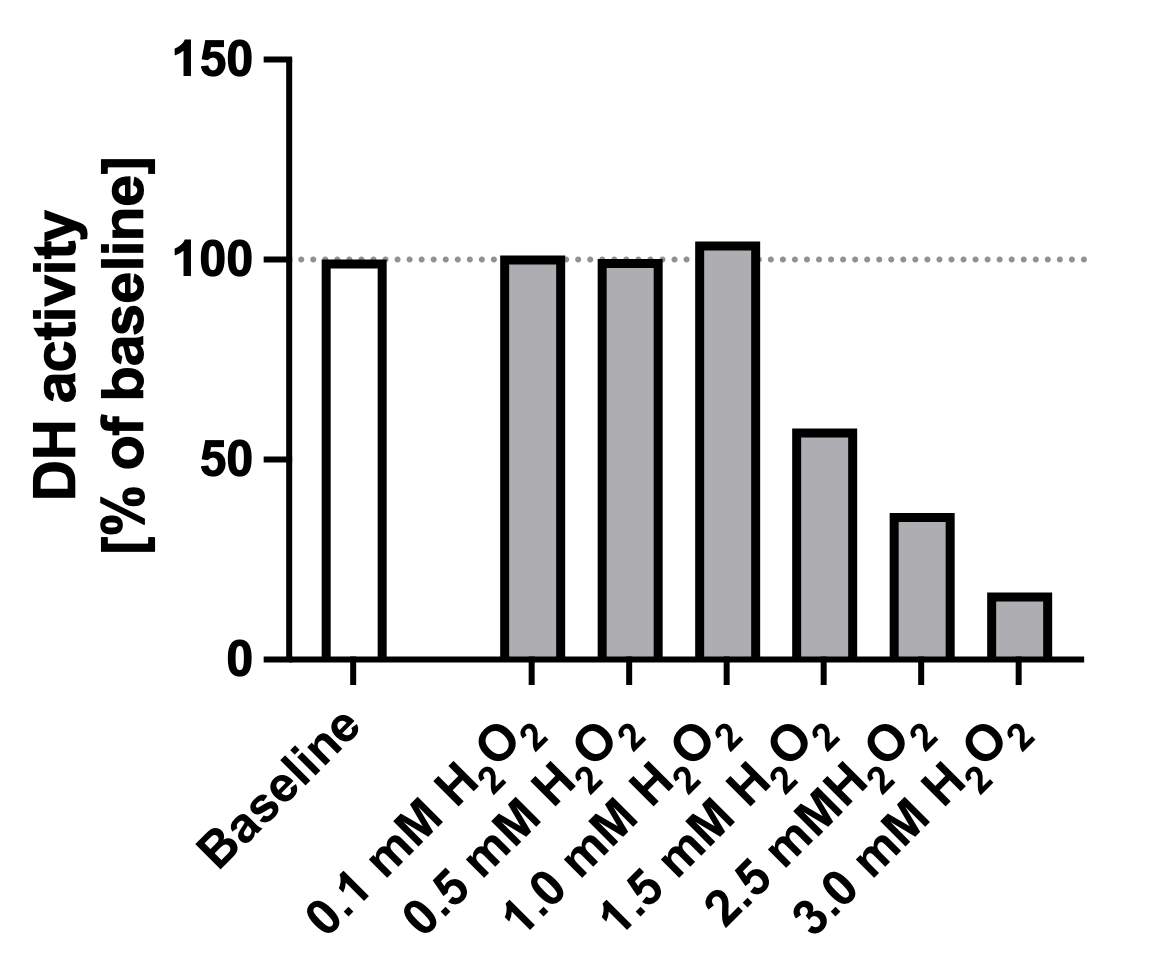

Supplement: Supplementary file 1 [file ijms-23-08292-s001.zip › Suppl Figure S2.tiff]

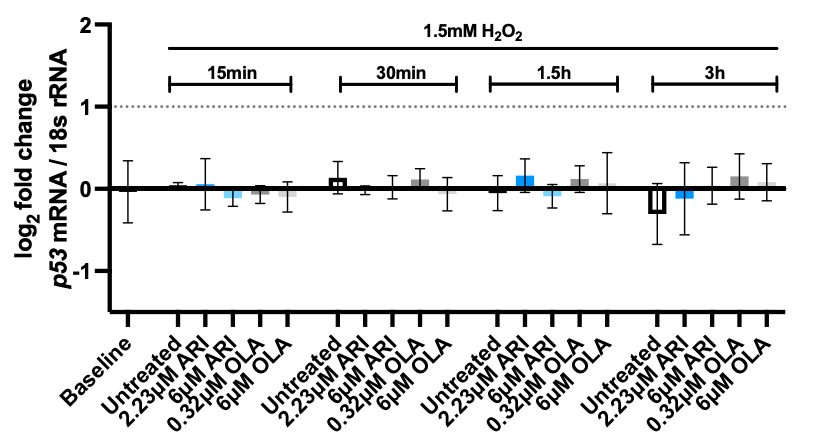

Supplement: Supplementary file 1 [file ijms-23-08292-s001.zip › Suppl Figure S3.tiff]

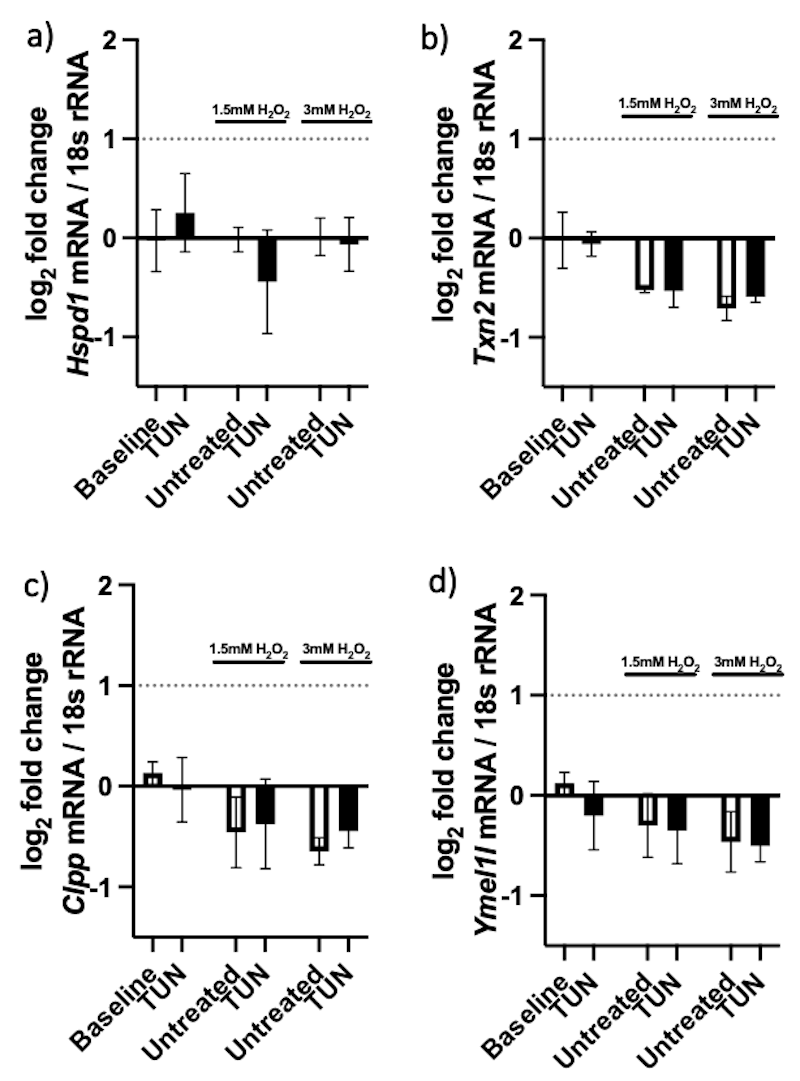

Supplement: Supplementary file 1 [file ijms-23-08292-s001.zip › Suppl Figure S4.tiff]

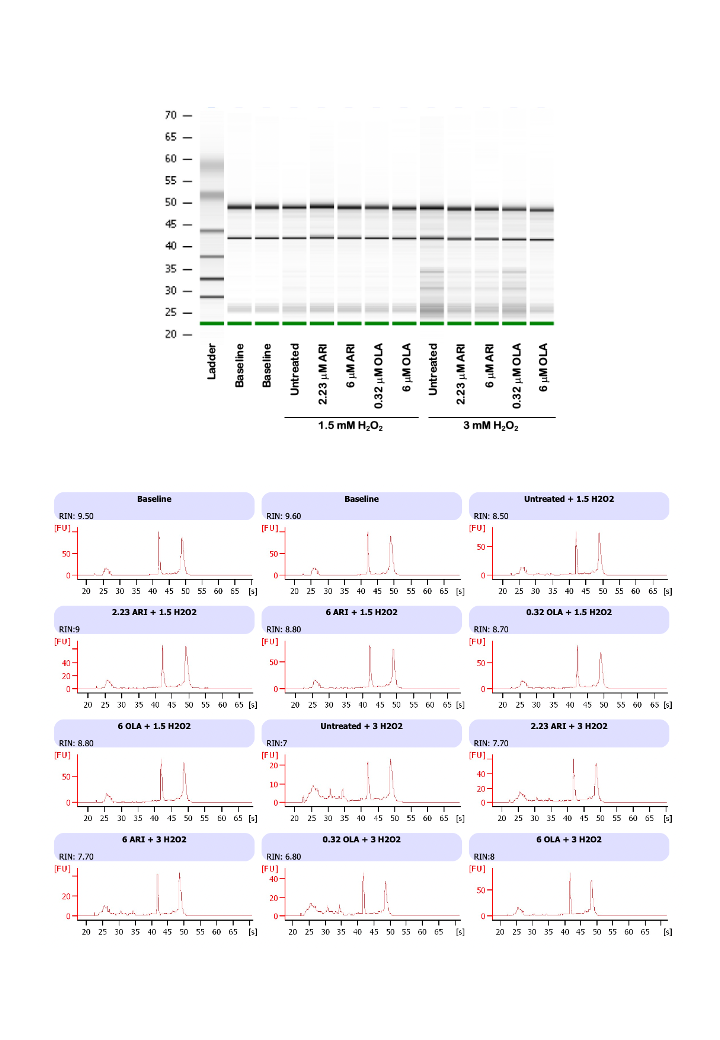

Supplement: Supplementary file 1 [file ijms-23-08292-s001.zip › Suppl Figure S5.tiff]

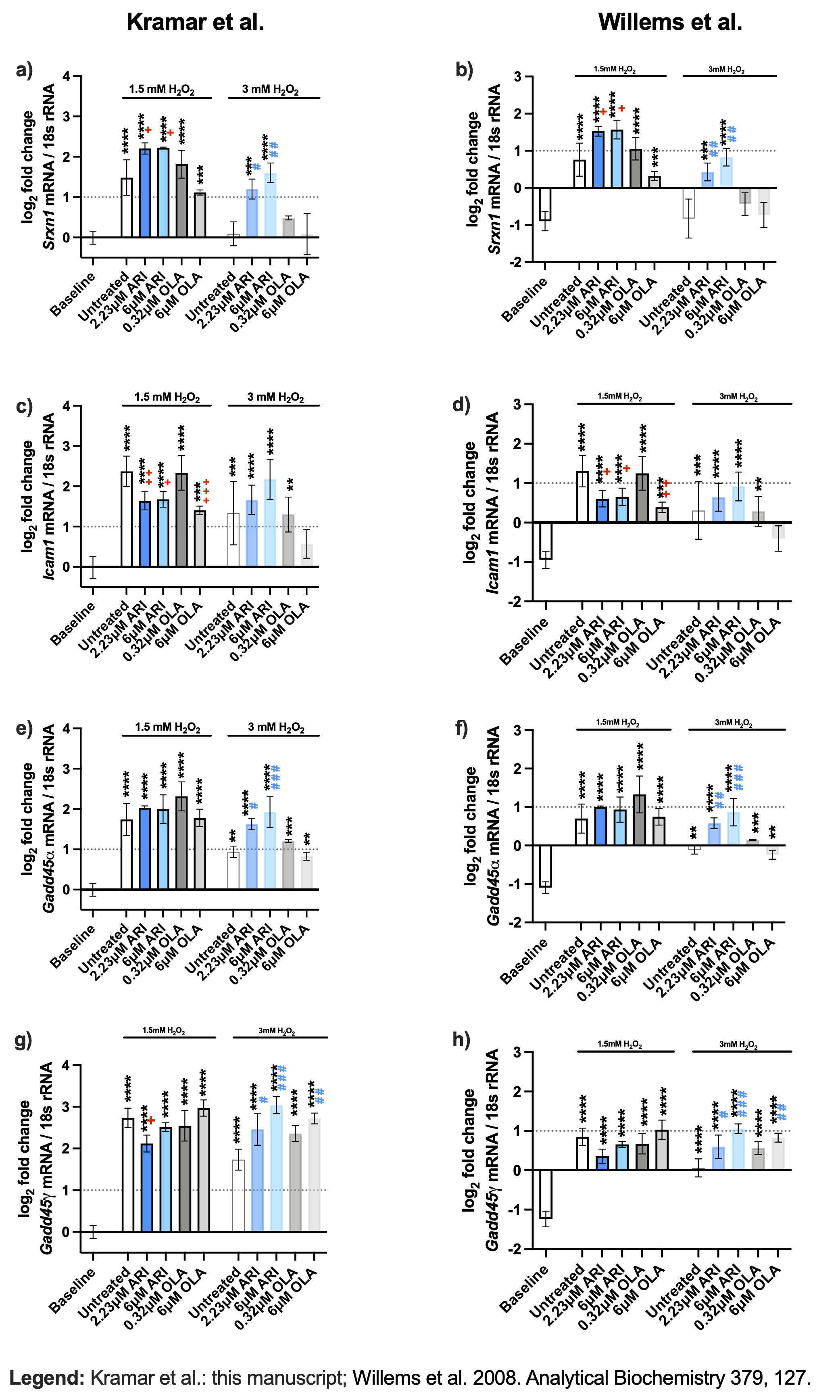

Supplement: Supplementary file 1 [file ijms-23-08292-s001.zip › Suppl Figure S6.tiff]
